# Editorial: Molecular mechanisms and therapeutic targets of cancer metastasis and therapy resistance

**DOI:** 10.3389/fonc.2025.1571403

**Published:** 2025-03-05

**Authors:** Ya-Wen Wang, Xu Chen

**Affiliations:** ^1^ Department of Breast Surgery, General Surgery, Qilu Hospital of Shandong University, Jinan, Shandong, China; ^2^ Department of Pathology, Qilu Hospital of Shandong University, Jinan, Shandong, China

**Keywords:** cancer metastasis, drug resistance, epithelial-mesenchymal transition, tumor microenvironment, combination therapies

Tumor metastasis and drug resistance are key factors that lead to cancer treatment failure and poor patient prognosis. Tumor metastasis refers to the spread of cancer cells from the primary tumor site to distant organs, where they form secondary tumor foci. The occurrence of metastasis involves complex cellular signaling pathways and changes in the tumor microenvironment (1). Tumor drug resistance, especially to chemotherapy, targeted therapy, and immunotherapy, significantly impacts treatment efficacy, resulting in recurrence and cancer progression. To improve the survival and quality of life of cancer patients, it is urgent to understand the molecular mechanisms of tumor metastasis and drug resistance and identify new therapeutic targets.

Tumor metastasis is a multi-step process, involving the detachment of cancer cells, invasion, dissemination through the blood or lymphatic systems, and growth in distant organs. Epithelial-Mesenchymal Transition (EMT) is a critical process through which cancer cells acquire invasive and metastatic potential (2, 3). The tumor microenvironment (e.g. cancer-associated fibroblasts, etc.) and immune cells (e.g. tumor-associated macrophages, etc.) also participate in tumor metastasis (4). Additionally, angiogenesis is an important condition for tumor metastasis. Deregulation of Cuproptosis has also been linked to tumor metastasis, and in a recent review by Wang et al., unbalanced levels of copper promote angiogenesis, enabling cancer cell spread. Moreover, tumor cells could degrade the extracellular matrix through the secretion of matrix metalloproteinases and other enzymes, creating conditions for crossing tissue barriers (5).

Some molecules and cell signaling pathways are also involved in the metastasis of tumor cells. Adhesion molecules (e.g. integrins, cadherins, etc.), growth factors and cytokines (e.g. epidermal growth factor, platelet-derived growth factor, etc.), Wnt/β-catenin pathway, PI3K/Akt/mTOR pathway play key roles in the metastasis of cancer cells (6, 7). Min et al. recently reviewed the impact of adhesion-associated molecule Desmoglein-2 (DSG2) on cancer cell adhesion, migration, invasion, angiogenesis, and vasculogenic mimicry. More importantly, they proposed that the pro-tumorigenic or anti-tumorigenic function of DSG2 is complex and context dependent.

Tumor drug resistance is a significant challenge in cancer treatment, as tumor cells employ various mechanisms to render therapeutic drugs ineffective or reduce their efficacy. P-glycoprotein, multidrug resistance-associated proteins family, and breast cancer resistance protein are examples of drug efflux pumps that expel chemotherapy drugs from tumor cells (8). Gene mutations including PIK3CA, KRAS, EGFR, p53 and OCT4 have been related to drug resistance phenotype (9). Tumor cells could undergo metabolic reprogramming or increasing antioxidant to enhance drug resistance (10, 11). DNA methylation, histone modification and noncoding RNAs regulation could alter gene expression leading to tumor cells escaping drug-induced death (12). Tumor cells could enhance DNA repair capabilities to resist DNA damage induced by chemotherapy drugs. Kaljunen et al. found that inactivation of the Fanconi anemia pathway could reverse prostate cancer drug resistance during DNA-damaging chemotherapy in a cell line-specific manner. Deregulation of apoptotic pathways would enhance cancer cells survival ability and lead to drug resistance (13). Tumor stem cells are a small population of cells within the tumor that possess self-renewal and multi-lineage differentiation capabilities, involving tumor drug resistance (14). High heterogeneity makes tumor cells vary in their sensitivity to chemotherapy, with some cells potentially harboring genetic mutations or phenotypic features related to drug resistance (15).

Researchers have proposed several potential therapeutic targets based on the molecular mechanisms of tumor metastasis and drug resistance. Targeting EMT-related molecules, inhibiting angiogenesis, intervening in extracellular matrix remodeling and inhibiting tumor microenvironment-related targets have shown promising therapeutic values for tumor metastasis. Clinically, using VEGF inhibitors (such as Bevacizumab) could block new blood vessel formation, thereby inhibiting tumor growth and metastasis. Inhibiting drug efflux pumps or modulating apoptosis pathways are also strategies to overcome tumor drug resistance. Therapeutic targets for tumor stem cells mainly focus on related signaling pathways (16). Due to the heterogeneity of tumor cells, treating tumor cell heterogeneity requires more precise strategies. By performing genetic testing and molecular typing of tumor cells, understanding the drug resistance-related gene mutations and phenotypic characteristics of tumor cells, targeted therapeutic drugs can be selected accordingly. Huang et al. reported a non-small cell lung cancer patient harboring a METex14 skipping mutation and with resistance to first-line tepotinib. Re-biopsy and reanalysis of the genetic profile of the tumor were performed, then second-line vebreltinib was administed and the patient achieved a prolonged response duration.

Immune checkpoint inhibitors (e.g. PD-1/PD-L1 inhibitors nivolumab, pembrolizumab, etc.), which enhance immune system attack on tumors, have been intensively investigated in several cancers (17). Immunotherapy targeting specific tumor antigens (e.g., HPV vaccines) boosts immune memory to prevent tumor recurrence (18). Taking tumor heterogeneity into consideration, combining multiple therapeutic approaches (such as chemotherapy with immunotherapy or targeted drugs with chemotherapy) has become an important strategy. Moreover, combining multiple drugs that target different pathways to provide comprehensive treatment against tumor cell heterogeneity is also a potential therapeutic strategy.

Tumor metastasis and drug resistance are two major challenges in cancer treatment. Currently, significant progress has been made in the research of therapeutic targets related to tumor metastasis and drug resistance. In the future, further research on the molecular mechanisms of tumor metastasis and drug resistance is needed to discover more effective therapeutic targets and develop more precise, efficient, and low-toxicity treatment strategies to improve the therapeutic outcomes and survival rates of cancer patients. Combination therapies, such as combining targeted therapies with chemotherapy, radiotherapy, and immunotherapy, may be an important direction to overcome tumor metastasis and resistance, and deserve further in-depth research and exploration. Additionally, with the continuous development of new technologies (such as CRISPR gene editing, single-cell sequencing), we could expect to obtain more precise therapeutic targets and strategies. By continuously delving into the molecular mechanisms of tumor metastasis and drug resistance, we may open up more effective treatment avenues, improving survival rates and quality of life for cancer patients.
